# Implementation of a Direct Oral Anticoagulants Interruption Protocol

**DOI:** 10.1055/a-2800-1414

**Published:** 2026-02-13

**Authors:** David Gimelfarb, Atefeh Ghorbanzadeh, Alfonso J. Tafur

**Affiliations:** 1 Department of Cognitive Science, Carleton College, Northfield, Minnesota, United States; 2 Internal Medicine Residency Program, Capital Health Regional Medical Center, Trenton, New Jersey, United States; 3 Division of Vascular Medicine, Endeavor Health, Skokie, Illinois, United States

Direct oral anticoagulants (DOACs), such as dabigatran, apixaban, edoxaban, and rivaroxaban, are widely used in clinical practice for the treatment of ischemic stroke in patients with atrial fibrillation (AF) as well as venous thromboembolism (VTE). Approximately 1 in every 10 chronically anticoagulated patients requires DOAC perioperative interruption. 1 Despite broad DOAC use, perioperative management lacks uniform guidance, particularly for VTE populations where normative data are scarce. Thus, perioperative management of DOAC varied widely among the practices used by different hospital systems and practices. 1 The implementation of a standardized DOAC interruption protocol has the opportunity to increase patient safety and case delivery consistency. 2 Additionally, the design of a guideline and algorithm for VTE patients would be significantly beneficial due to the paucity of normative data available on VTE.

This prospective, observational, implementation study aimed to illustrate the process of standardization and implementation of the DOAC interruption protocol in a multidisciplinary practice, anchored in change management tools used across a multihospital system.

## Methods

**Fig. 1:**
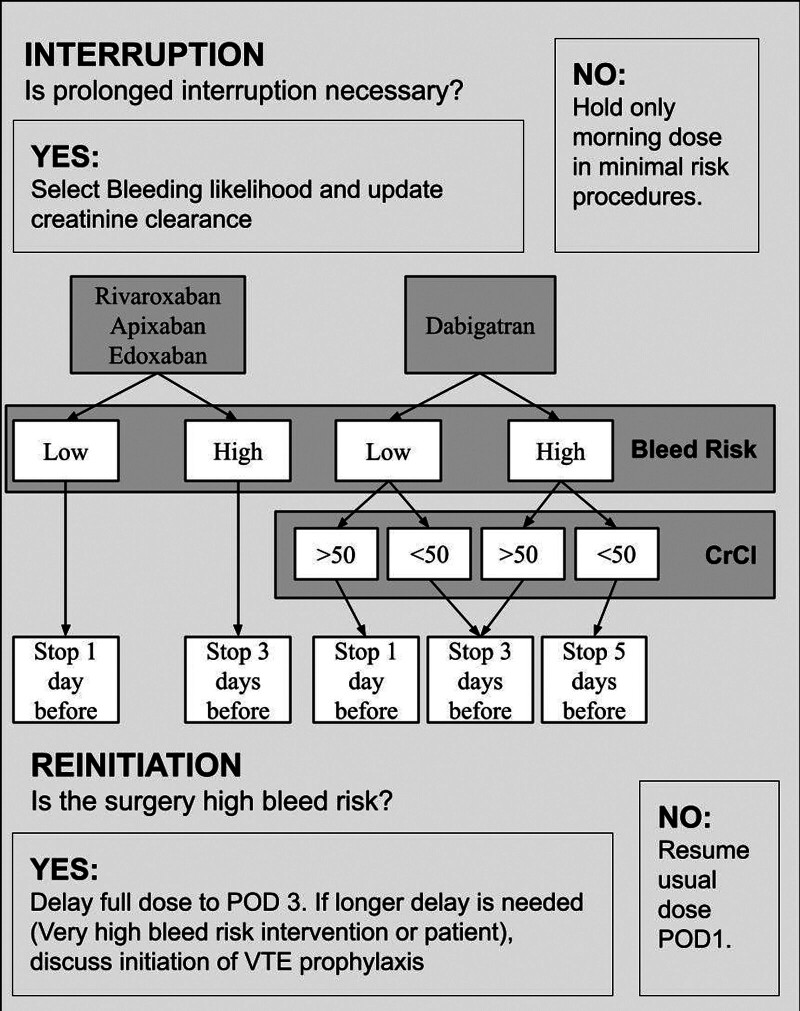
DOAC interruption protocol algorithm adapted from PAUSE and ASRA guidelines, showing decision pathways based on DOAC type, bleeding risk, and creatinine clearance levels. VTE, venous thromboembolism.

We conducted this study across four Endeavor Health hospitals by using Kotter's eight-step change model to create systemic change within an organization. This model outlines creating urgency by identifying problems, forming a coalition of leaders with a vision for change, communicating, testing, and iterating upon the vision, and anchoring change within organizational culture. 3 Our multidisciplinary team included hematologists, vascular specialists, cardiologists, anesthesiologists, pharmacists, and quality leadership. First, we developed the interruption protocol and algorithm through a process of communication, pilot testing, and iterative reinforcement. Following the literature summary, we used the Delphi method 4 to generate stakeholder consensus. The coalition developed an adapted version of the Perioperative Anticoagulant Use for Surgery Evaluation (PAUSE) protocol 5 and the American Society of Regional Anesthesia (ASRA) guidelines 6 ( Fig. 1 ). The revised protocol examines factors such as DOAC type, bleeding risk, and creatinine clearance level. Depending on these variables, patients were classified with a DOAC interruption timing ranging from days 1, 3, or 5 prior to surgery. Following the steps of the Kotter model, our team disseminated the algorithm to registered nurses working in departments such as vascular medicine, hematology, and cardiology, to ensure the algorithm was established.

Only elective procedures were eligible for inclusion; cases involving emergent surgery or patients with intramuscular/neuraxial procedures were excluded.

After a lead time, we prospectively collected patient demographics and tracked 3-month outcomes, specifically monitoring for major bleeding and thrombotic events following elective procedures. Outcomes were defined as arterial events, subcategorized into ischemic stroke, transient ischemic attack (TIA), acute myocardial infarction, or unclassified arterial event, venous events, subcategorized into new distal/proximal deep vein thrombosis and pulmonary embolism, and major bleeding. Major bleeding was defined per International Society on Thrombosis and Haemostasis (ISTH) criteria as bleeding that is fatal, occurs in a critical organ or area such as intracranial, intraspinal, or pericardial, causes a fall of hemoglobin level of 20 g/L or more, and/or a surgical site that requires a second intervention or interferes with rehabilitation significantly. Reference data were collected from October 2019 to June 2021.

This study was approved by the Endeavor Health Institutional Review Board (Eh19-243). Individual patient consent was waived due to the observational nature of the study.

## Results

We summarize outcomes in 301 chronically anticoagulated patients on DOAC undergoing perioperative interruption. The study population had a mean age of 74 years (SD 10.3), with 174 (57.8%) male patients. Nearly all procedures (97.7%) were classified as low bleeding risk.

Most patients (85.7%) had AF as their primary indication for anticoagulation, with persistent AF being the most common subtype (65.5%). VTE was present in 76 patients (25.3%). We stratified patients into three groups: VTE-only ( *n* = 34), AF-only ( *n* = 216), and patients with both conditions ( *n* = 42). Major bleeding events were rare overall, occurring in 1.4% of AF-only patients and 2.4% of patients with both conditions, while no major bleeding occurred in VTE-only patients. Arterial thromboembolic events occurred in 1.4% of AF-only patients and 2.4% of patients with both VTE and AF. Among AF patients, ischemic stroke occurred in 0.92% and TIA in 0.46%. No arterial events were observed in VTE-only patients. Venous thromboembolic events were uncommon, occurring only in VTE-only patients (2.9%), specifically as pulmonary embolism. No new venous events occurred in the other patient groups.

## Discussion

The combined stroke/TIA rate of 1.38% in our AF patients compares favorably with previously reported rates of 0.16% to 0.6% in similar perioperative populations. 2 There is a paucity of comparative data on VTE outcomes. Comparisons to literature are limited, given the rate of non-major procedures dominated investigations. Our study was limited by a lack of use of interventional radiology data, and the process was not fixed to a protocol. Subsequent follow-up of sustained adoption of the interruption protocol was also not confirmed.

Our model demonstrates how structured change management facilitated consistent DOAC interruption practices across four hospitals, reducing variation without excess bleeding or thrombosis. Though we achieved implementation of change on a systemic level, additional efforts are needed to ensure long-term sustainability. We illustrate a Delphi method approach and the Kotter implementation model as potential tools to reach interdisciplinary agreement in perioperative anticoagulation, where guidelines remain conflictive.
